# Learning mitigates genetic drift

**DOI:** 10.1038/s41598-022-24748-8

**Published:** 2022-11-27

**Authors:** Peter Lenart, Julie Bienertová-Vašků, Luděk Berec

**Affiliations:** 1 Faculty of Science, Research Centre for Toxic Compounds in the Environment, Masaryk University, Kamenice 5, Building A29, 62500 Brno, Czech Republic; 2 Department of Experimental Biology, Faculty of Science, Masaryk University, Kamenice 5, 62500 Brno, Czech Republic; 3 Faculty of Science, Institute of Cell Biology, University of Bern, Baltzerstrasse 4, 3012 Bern, Switzerland; 4 Department of Mathematics, Faculty of Science, Centre for Mathematical Biology, University of South Bohemia, Branišovská 1760, 37005 České Budějovice, Czech Republic; 5 Department of Ecology, Biology Centre, Institute of Entomology, The Czech Academy of Sciences, Branišovská 31, 37005 České Budějovice, Czech Republic

**Keywords:** Evolution, Evolutionary genetics, Evolutionary theory, Population genetics, Genetic variation

## Abstract

Genetic drift is a basic evolutionary principle describing random changes in allelic frequencies, with far-reaching consequences in various topics ranging from species conservation efforts to speciation. The conventional approach assumes that genetic drift has the same effect on all populations undergoing the same changes in size, regardless of different non-reproductive behaviors and history of the populations. However, here we reason that processes leading to a systematic increase of individuals` chances of survival, such as learning or immunological memory, can mitigate loss of genetic diversity caused by genetic drift even if the overall mortality rate in the population does not change. We further test this notion in an agent-based model with overlapping generations, monitoring allele numbers in a population of prey, either able or not able to learn from successfully escaping predators’ attacks. Importantly, both these populations start with the same effective size and have the same and constant overall mortality rates. Our results demonstrate that even under these conditions, learning can mitigate loss of genetic diversity caused by drift, by creating a pool of harder-to-die individuals that protect alleles they carry from extinction. Furthermore, this effect holds regardless if the population is haploid or diploid or whether it reproduces sexually or asexually. These findings may be of importance not only for basic evolutionary theory but also for other fields using the concept of genetic drift.

## Introduction

Genetic drift is a major evolutionary process characterized by random fluctuations of allele frequencies in a population from one generation to the other. Such random fluctuations can be big enough to trigger allele fixation and loss of alternative alleles. It is generally thought to depend on the population size, the number of reproducing individuals, and variance in reproductive success^1–3^. The key concept used to quantify the effect of drift on real-world populations is the effective population size introduced by Sewall Wright in 1930s^4,5^. While there are many extensions of Wright`s work allowing calculation of the effective population size under different ecological scenarios^3^ or with different reproductive behaviors^6^, none of them considers the effects of non-reproductive behaviors and the history of a given population. Just as in other fields of biology^7,8^ such a simplification has proven to be very useful, but as we show further in this article, it is likely incomplete. Indeed, just as a phenotype of an individual animal may change and develop during its life, so too can it change its response to various potentially deadly situations. This is crucial because if such a change is systematic (i.e., not random), it can change how a random source of mortality affects allelic numbers even when the overall mortality rate at the population level remains the same.

Let us imagine a hypothetical scenario. Consider a species subject to predation, with all individuals genetically identical so that there is no selection acting upon them. Every young individual of this prey species has the same chance to escape a predator if it is attacked, say 50%. However, if an individual survives a predator’s attack, it learns from this experience and is more cautious or skillful in the future, increasing its chances to survive a future encounter of a similar kind (e.g., to 75%). Moreover, every future attack that the prey individual survives allows it to learn from it and further increases its chances to survive the next one (albeit at a decelerating rate). In other words, individuals which survive and learn from such attacks increase their fitness by gaining an advantage compared to those which have not yet encountered predators, even though this advantage has random origins and is not heritable. As a consequence, alleles carried by a lucky individual get a temporary boost. It is thus less likely that these alleles are removed from the population. Furthermore, because such an effect is random, many alleles benefit at some point, thus slowing the loss of genetic diversity.

The scenario with the prey learning to escape predators is just one specific example, and the same principle can be applied to other situations and effects. For example, a population affected with a deadly pathogen in which individuals that survive infection increase their immunity should show the same trend. In this article, we test just this hypothesis that a systematic increase of organisms` chances of survival (e.g., learning, immunological memory) in response to a random source of mortality can mitigate genetic drift (slower loss of alleles) even when the mortality rates for both learning and non-learning scenarios remain the same. For this purpose, we develop an agent-based model that monitors allele frequencies in time in a population of prey, either able or not able to learn how to avoid or escape predators more effectively from the experience of unsuccessful past predator attacks. Both of these populations start with the same size and have the same overall mortality rates. The main focus of our analysis is on the number of alleles and their loss in learning and non-learning scenarios.

## Methods

The agent-based model developed for this article monitors allele frequencies in time in a population of prey, either able or not able to learn how to avoid or escape predators more effectively from the experience of unsuccessful past predator attacks. It has these properties:

1. There are no mutations, migration, or selection in this model. There are no functional differences between individuals or alleles they carry. Alleles have no effect on the individuals carrying them. This is intentional as we aim to study the effect of learning on the genetic drift in isolation from other evolutionary forces. For the same reason, the default version of the model does not consider the effects of aging.
2. At the start of the simulation, population size and every other parameter except the ability to learn are the same for learning and non-learning populations.
3. Overall mortality rates due to predation are constant and the same for both learning and non-learning populations. This is not affected by the fact that prey individuals in the learning population have, on average, a higher chance to escape predator attacks because predators repeat their attacks until a given number of prey individuals are killed. Therefore, predators may need to repeat their random attacks more times in the learning population; however, in the end, the same number of prey die each timestep in both the learning and non-learning population.
4. If not stated otherwise, generations overlap.

The precise workings of the models are as follows:

We start with a given number of prey individuals (1000) and an initial number of unique alleles (100). The individuals are assumed haploid and each obtains a randomly chosen allele. Moreover, each individual has a probability of avoiding or escaping the predator’s attack (initially 0.5). Each simulation runs for a given number of time steps (100). Predation is implicit: each time step, a given number of prey individuals are removed from the population—an individual is randomly selected, and its probability to avoid or escape predation is used to decide whether the predator’s attack is successful. If yes, the individual is removed from the population. This random selection of prey is repeated until a given number of prey are removed from the population. The number of removed prey individuals per time step is always the same between the learning and non-learning scenario. If the attack is not successful and prey are learning, we distinguish accelerating and decelerating rates of learning. In the former case, the new value of escape probability is the old value increased by a given proportion of that old value, limited by a preset maximum value (0.99). In the decelerating case, the new escape probability value is the old value increased by a given proportion of the difference between a present maximum value and the old value. In the default setting, an update in the probability of escaping predation happens at the end of the timestep regardless whether an individual prey survived one or multiple predator attacks. In an alternative setting, learning occurs after every unsuccessful attack. In addition, there is an upper bound on the probability of avoiding or escaping predation. Finally, the removed prey individuals are replaced by new ones: at the end of the time step, a surviving individual is randomly chosen (with repetition) and gives birth to one offspring until the population is restored to its original size (1000). Each offspring inherits the allele from its parent, and its probability of avoiding or escaping the predator’s attack is set to the initial value (0.5).

The haploid sexual and diploid sexual variations of the model differ from the above description only in the way reproduction is carried out. In the model with haploid sexually reproducing individuals, following predation, mating pairs are formed randomly and produce one offspring, its allele is randomly taken from one of its parents. In the model with diploid sexually reproducing individuals, each individual has two alleles, randomly chosen initially from the same set of 100 alleles as considered for haploid individuals. Following predation, mating pairs are again formed randomly and produce one offspring; its alleles are randomly taken from the respective pairs of alleles of its parents.

With this model, we have tested how the varying number of removed prey, varying upper bounds of learning effectiveness, default vs. immediate learning, accelerating and decelerating rate of learning, limits in maximal age of prey, and asexual vs. sexual reproduction affect the ability of learning to decelerate loss in the number of alleles.

To help understand potential differences between the learning and non-learning scenarios, we calculate the generation time, defined as the mean time interval between the birth of an individual and the birth of its offspring. Within our agent-based model, we calculate it as follows. We follow each newborn through all its life, establish a variable for it, and for each offspring it has, we add to this variable the age at which that offspring was produced. After an individual dies, we divide this variable by the total number of offspring this individual produced during their life. This individual generation time is then averaged over all individuals born in all simulations pertaining to a specific scenario to get the population generation time.

The above-described model was run using statistical software R, v. 4.0.1.

## Results

### The haploid asexual model

**Figure 1:**
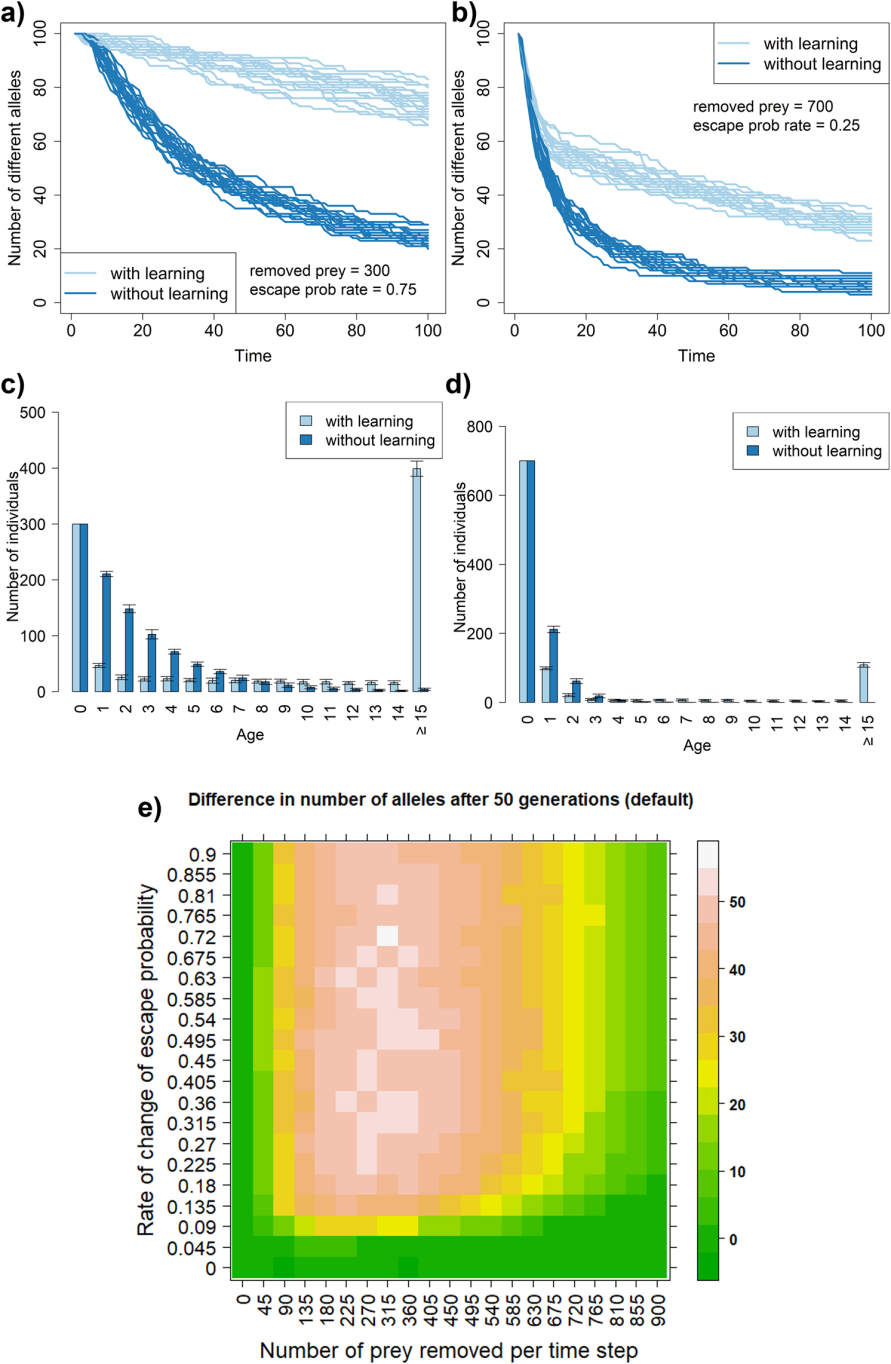
Intensity of predation modulates interaction of learning and genetic drift. Temporal dynamics of the number of alleles present in populations of prey subject to predation. Panels (**a**) and (**b**) show 20 simulation runs for both learning and non-learning scenarios. Panels (**c**) shows age distributions for panel (**a**) and panel (**d**) show age distribution for panels (**b**). The values in the heatmap are averages from 10 simulation runs. All prey populations consist of 1000 individuals at the beginning of each time step. Individuals killed by predators are replaced at the end of each step through reproduction of surviving individuals ("Methods"). Predators kill the same number of individuals in both learning and non-learning scenarios to allow for a fair comparison. The upper bound on the probability of avoiding or escaping a predator attack is set to 99%. The learning occurs in a default setting, i.e., at the end of every timestep.

First, we tested the hypothesis that learning mitigates genetic drift on a simple model assuming haploid, asexually reproducing prey ("Methods"). Simulation results clearly show that ability to learn from experience significantly reduces the loss of alleles caused by genetic drift (Fig. 1a,b and Supplementary Fig. S1). On average (from 20 simulations) after 100 timesteps 12.4 (SD = 1.14) and 50.2 (SD = 2.77) alleles remain in non-learning and learning scenarios, respectively. To allow fair comparison, learning and non-learning populations in our model start with the same population size and have the same overall mortality rates (the same number of prey is removed from the population at each time step in any scenario), and produce the same number of offspring (equal to the number of removed individuals—this keeps population size constant over time). The slower loss of genetic variability in a learning scenario is caused by the creation of a pool of experienced individuals less likely to die, thus protecting the alleles they are carrying from extinction (Fig. 1c,d).

The size of this effect depends on specific circumstances. Our simulations consistently showed that the effect of learning on the genetic drift was affected by the fraction of the population killed by predators as well as by the learning rate (Fig. 1e). In addition, the effect of learning is surprisingly strong. In some parameter settings, it can lead up to 50 alleles difference between learning and non-learning scenarios after a mere 50 generations. This is a considerable difference given that both scenarios start with 100 unique alleles.

**Figure 2:**
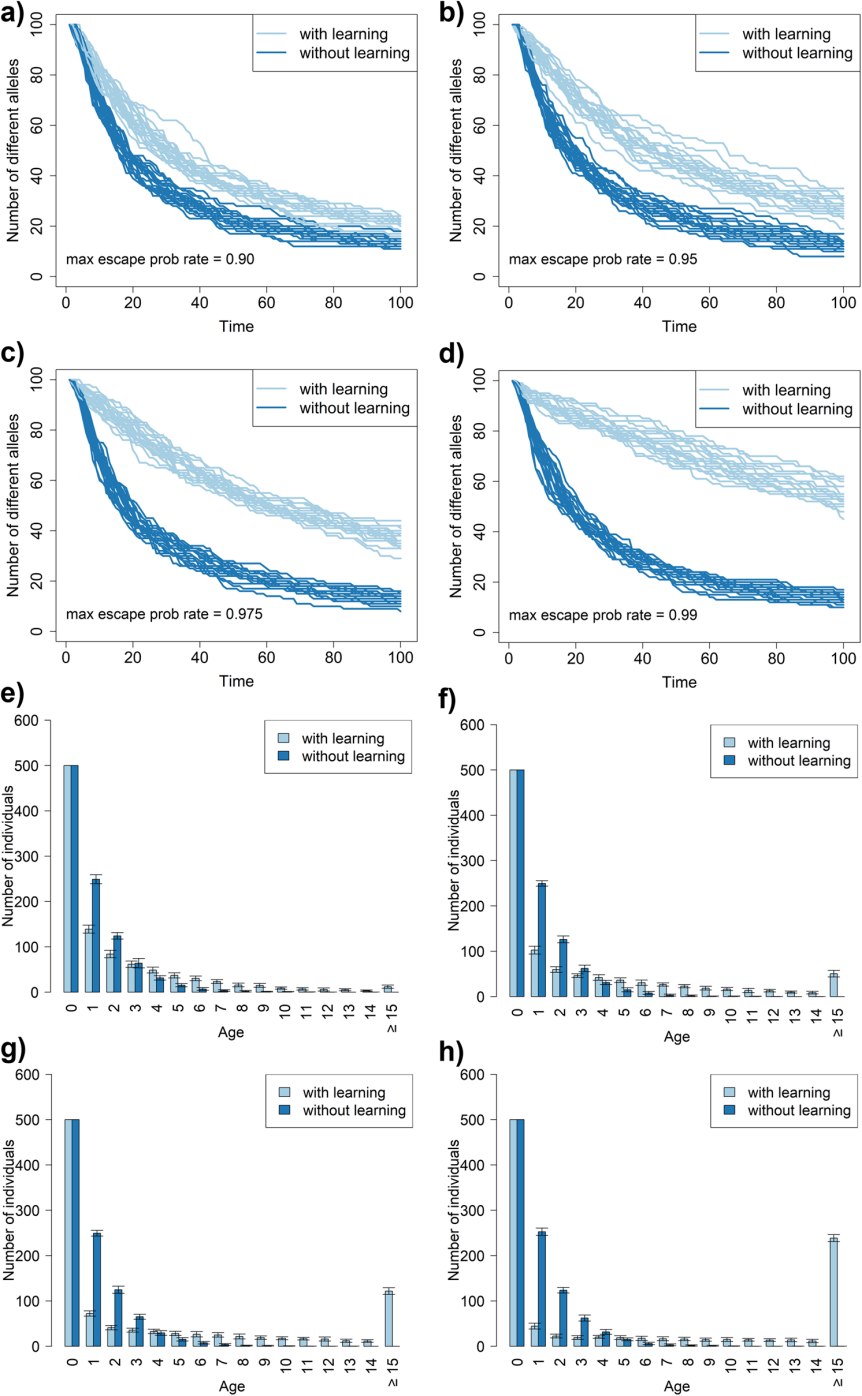
Effectiveness of learning dictates its effect on genetic drift. Temporal dynamics of the number of alleles present in populations of prey subject to predation. Panels (**a**–**d**) show different settings on the upper bound on the probability of avoiding or escaping predator attack. Panels (**e**–**h**) show age distributions for panels (**a**–**d**) (in order). All prey populations consist of 1000 individuals at the beginning of each time step. Individuals killed by predators are replaced at the end of each step through the reproduction of surviving individuals ("Methods"). Predators consume 500 prey individuals per time. Predators kill the same number of individuals in both learning and non-learning scenarios to allow for a fair comparison. Each panel shows 20 simulation runs for both learning and non-learning scenarios.

The ability of learning to conserve genetic diversity also depends on how effective learning is (Fig. 2). In our simulations, the maximal limit on how effectively prey could learn to avoid or escape predators was an even more important factor than the intensity of predation itself. When prey could learn at best to escape a predator attack with 90% probability, the effect of learning was noticeable (Fig. 2a) but much smaller than when they could eventually reach 95% chance (Fig. 2b), 97,5% chance (Fig. 2c) or even 99% chance to escape (Fig. 2d). Note that in all cases, the effect of learning could be explained by the systematic creation of a fraction of long-lived individuals (Fig. 2e–h).

**Figure 3:**
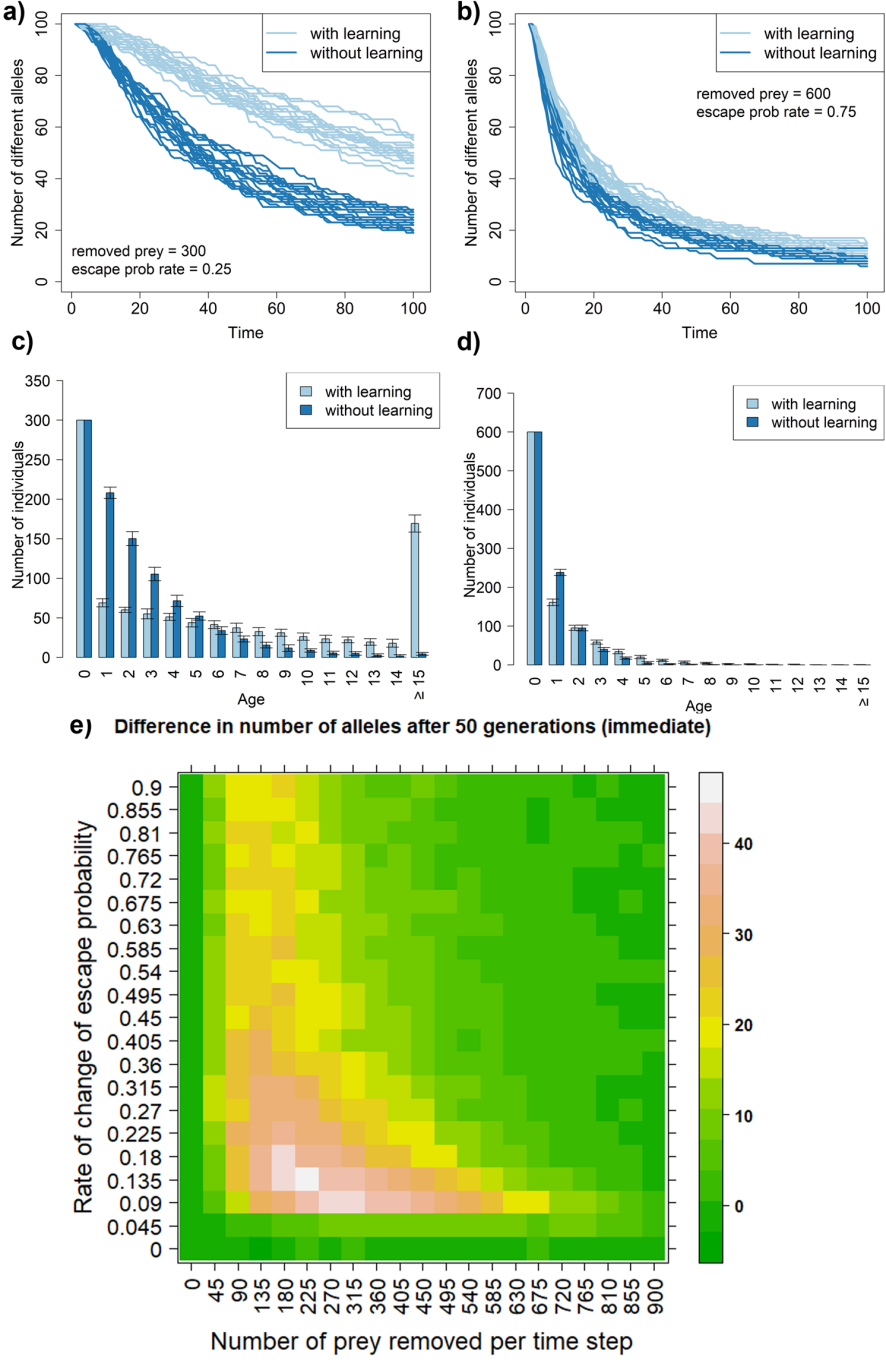
Immediate learning affects drift in a distinct way from stepwise learning. Temporal dynamics of the number of alleles present in populations of prey subject to predation. Panels (**a**) and (**b**) show 20 simulation runs for both learning and non-learning scenarios. Panels (**c**) shows age distributions for panel (**a**) and panel (**d**) show age distribution for panels (**b**). The values in the heatmap are averages from 10 simulation runs. In contrast to previous simulations prey learns instantly after unsuccessful predators` attacks. All prey populations consist of 1000 individuals at the beginning of each time step. Individuals killed by predators are replaced at the end of each step through a reproduction of surviving individuals ("Methods"). Predators kill the same number of individuals in both learning and non-learning scenarios to allow for a fair comparison. The upper bound on the probability of avoiding or escaping a predator attack is set to 99%.

Learning after every unsuccessful attack instead of at the end of every timestep produces qualitatively distinct results (Fig. 3) that, nevertheless, support the hypothesis that learning mitigates genetic drift. Still, the effect remains strong as it can lead to a difference of up to 50 alleles in 50 timesteps.

Learning could operate in various ways. For example, prey could learn at an accelerating or decelerating rate. Thus, we have tested how these distinct learning modes affect the ability of learning to decelerate the loss of alleles. While there were differences between these two modes they were limited. The default setting of accelerating learning (i.e., every succeeding learning gives greater survival benefit) produces qualitatively analogous results as the setting with decelerating learning (i.e., every succeeding learning gives smaller survival benefits); see Supplementary Figure S2.

At first glance, it seems likely that the mean lifespan could increase in the learning scenario, which could potentially increase effective population size, thus, decreasing genetic drift. Interestingly, the mean lifespans in learning and non-learning scenarios are almost identical in our simulations (Table 1). In addition, the tiny difference between the mean lifespan that occurs in our scenarios are most often in the opposite direction (i.e., mean lifespan) in the learning scenario is slightly shorter. Nevertheless, the learning scenario consistently shows higher variability in lifespan (Figs. 1, 2 and 3).

**Table 1:** Learning scenario does not increase the mean lifespan.

No_remove	Immediate_update	Learning		Non-learning	
		Mean lifespan	SD	Mean lifespans	SD
200	F	7.3	13.6	7.3	13.4
500	F	2.8	9.6	3.0	8.1
800	F	1.8	6.8	1.9	6.4
200	T	7.1	14.7	7.3	13.4
500	T	3.0	8.4	3.0	8.2
800	T	1.9	6.5	1.9	6.4

Mean lifespans and SD are calculated from 10 replicates by pooling them together.

**Figure 4:**
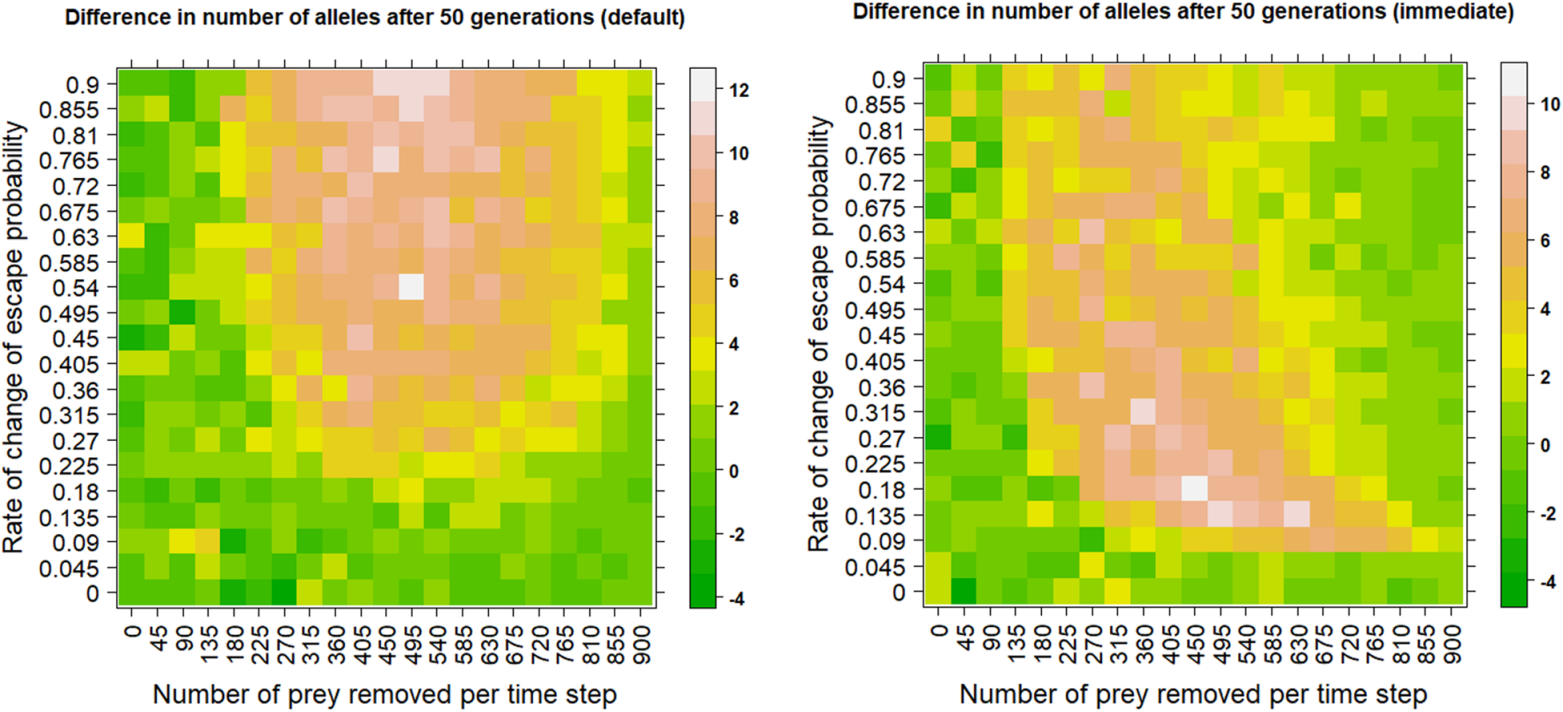
Restriction of maximal possible age makes effects of learning smaller but still noticeable. The heatmaps show the result of simulations with the capped maximum age of 10. Every individual that passed this threshold instantly died. Predators kill the same number of individuals in both learning and non-learning scenarios to allow for a fair comparison. The upper bound on the probability of avoiding or escaping a predator attack is set to 0.99. The values in the heatmap are averages from 10 simulation runs. The learning occurs at the end of each timestep in the left panel and after every unsuccessful predator attack in the right panel.

While the mean lifespan does not vary much between learning and non-learning scenarios, the distribution of ages is substantially different, with older individuals being overrepresented in the learning scenario (Figs. 1, 2 and 3). This demonstrates that average values are not representative of the situation. However, learning still slows down the decrease in the number of alleles even with introducing a fixed maximal age, after which every individual dies (Fig. 4).

**Figure 5:**
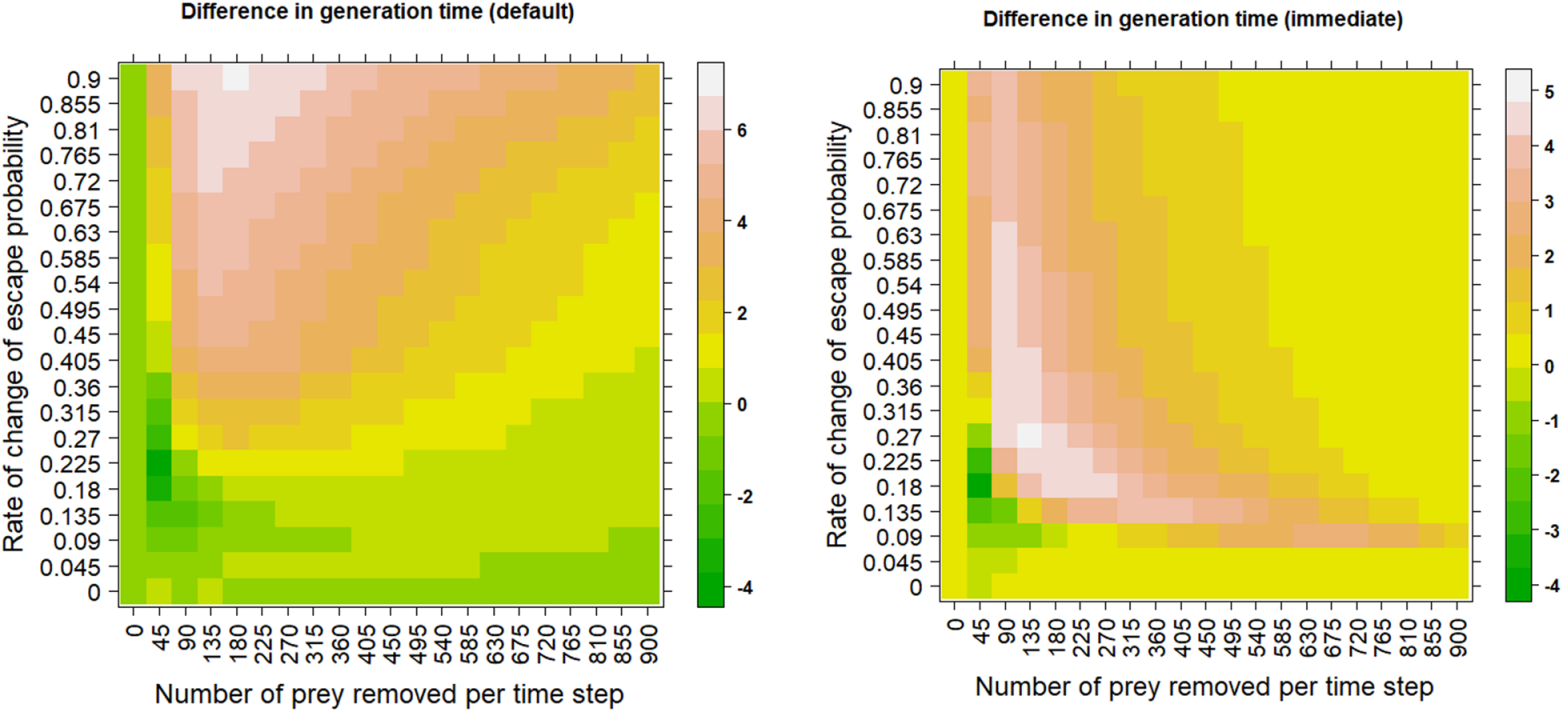
Learning often prolongs generation time. The heatmaps show the mean difference in generation time between learning and non-learning scenarios. Predators kill the same number of individuals in both learning and non-learning scenarios to allow for a fair comparison. The upper bound on the probability of avoiding or escaping a predator attack is set to 0.99. The values in the heatmap are averages from 10 simulation runs. The learning occurs at the end of each timestep in the left panel and after every unsuccessful predator attack in the right panel.

More importantly, while the generation time is higher for learning scenarios in most settings (Fig. 5), there is no difference between learning and non-learning scenarios in some settings. For example, the simulations with the rate of change of escape probability of 0.09 and the number of prey removed from 135 to 360 with the default mode of learning (after each step) seem to have no difference in generation time. Note that in this parameter range, there is still a substantial difference in the loss of alleles between learning and non-learning scenarios (Figs. 1 and 3).

### The diploid sexual model

**Figure 6:**
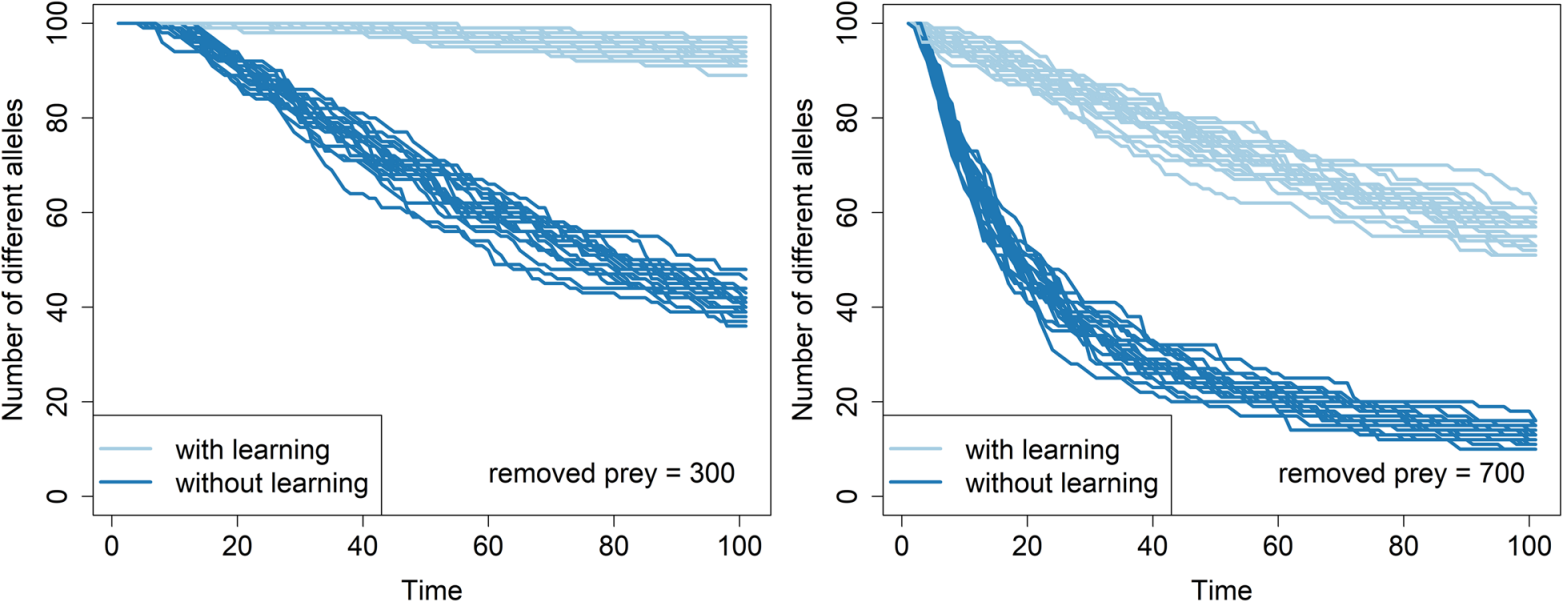
Effect of learning on genetic drift in *a* diploid sexually reproducing population. Temporal dynamics of the number of alleles present in populations of prey subject to predation. All prey populations consist of 1000 individuals at the beginning of each time step. Individuals killed by predators are replaced at the end of each step through reproduction of surviving individuals ("Methods"). In panel (**a**), predators consume 500 prey individuals per time step, while in panel (**b**), predators consume 800 prey individuals per time step. Predators kill the same number of individuals in both learning and non-learning scenarios to allow for a fair comparison. The upper bound on the probability to avoid or escape predator attack is set to 99% for both panels. Each panel shows ten simulation runs for both learning and non-learning scenarios.

Finally, we created a version of the model with diploid sexually reproducing prey individuals ("Methods"). The results of this model show once again that learning from experience significantly reduces genetic drift (Fig. 6). The main difference in comparison to the results of haploid model versions is that, as could be expected, genetic drift reduces genetic diversity at a slower pace.

## Discussion

Genetic drift describes random fluctuations in allelic diversity in a population. In this article, we have reasoned that a systematic increase of organisms` chances of surviving in response to a random source of mortality, e.g., learning from experience, mitigates genetic drift (decrease in the number of alleles) even when overall mortality rates remain the same. To test this hypothesis, we developed an agent-based simulation model. The results of our simulations show that even though the same number of prey die per time step in both the learning and non-learning scenarios, their allele numbers decrease at quite different rates. This effect is explained by the tendency of learning to create a pool of older experienced individuals who have a very small chance of dying from predation and thus create a pool of alleles that are unlikely to be lost. In addition to confirming our hypothesis in general, our results also demonstrate that this effect applies regardless of whether the affected organism is haploid and reproduces asexually or diploid and reproduces sexually.

Our results suggest that learning from experience may be an overlooked factor affecting effective population size. Contrary to most other factors such as an unequal number of males and females^4,9,10^, the ability to learn from the experience possibly increases the effective population size.

The purpose of this article is to point at the existence of a complex relationship between various mechanisms improving organisms’ ability to survive and the strength of genetic drift. The results presented here cannot, obviously, be expected to tell us anything about how strong this effect is in nature. With regard to learning from experience, the crucial factor for determining this would be assessing how effectively animals can learn to avoid or escape a deadly danger. For simulations in this article, we operated with an efficiency of 90% or more. Arguably, this probability may be too high in real-world settings. However, under certain circumstances, it is plausible. For example, a study on barn owl (*Tyto alba*) has shown that these owls have a 90% chance of catching a stationary food item and only a 21% chance of catching moving items^11^. Furthermore, the owls were even less likely to catch an item moving towards them (18%), and most interestingly, if the food items were moving sideways the barn owls were not able to catch them at all. Therefore, it seems feasible that prey may learn simple strategies that would allow them to escape at least some types of predators with surprisingly high probability. Nevertheless, even precise information about how effectively animals can learn to avoid or escape different sources of mortality may not be enough to predict the effect of learning on the genetic drift in a real-world population because the ability to learn is by itself an adaptive trait and including selection into the model would further complicate interpreting its results. One way or another, our message remains the same; predicting the strength of genetic drift is more complex than conventionally assumed.

At first glance, it may seem that the effect of learning presented in this article is caused merely by the fact that learning increases generational interval (the average age of parents when their offspring are born), which is known to result in increased effective population size and thus smaller genetic drift^10^. However, as we have shown, while learning increases generation interval in most simulation settings, there are situations where it decreases loss in the number of alleles without prolonging generation interval. Therefore, the lengthening of generation intervals likely explains part but not the entire effect of learning. However, this is only because the generation interval is an average metric that cannot reflect changes resulting from changes in age distribution that do not change average values. This can occur in some settings with learning scenarios as learning not only creates a pool of older hard-to kill individuals but also decreases the number of individuals surviving up to some age (simulation specific);see Fig. 1c,d.

We exemplified our idea of learning mitigating genetic drift by considering the effect of learning in the context of predation. However, for the same reasons, learning exerts the same influence on genetic drift caused by any source of mortality, which individuals of a given species may learn to avoid. For example, if individuals of a given species were able to learn to avoid traffic from their experience, the effect on genetic drift would be identical to the learned ability to avoid or escape predators. To go even further, any learned improvement in the ability to find a partner for mating, to find food and thus decrease the risk of starvation, or to care for offspring and thus increase their chance of reaching adulthood, will, to some extent, help preserve genetic diversity despite the raw effects of drift. Accordingly, there may be other types of reactions, such as immunological memory, which could help preserve genetic diversity by mitigating genetic drift through the same principle.

It is important to note that our model assumes overlapping generations, and thus all our results apply only to such a situation. With nonoverlapping generations, the benefits of nonhereditary learning are likely to be nonexistent or much smaller as there is much less time for them to have any effect (provided that learning is immediate). In our scenarios with overlapping generations, individuals that got lucky and learned to evade predators effectively could reproduce several times before their death. With nonoverlapping generations, they would be able to reproduce only once regardless of how well they can escape predators which would undoubtedly reduce the benefits of learning.

The here-described relationship between learning and genetic drift also leads to some testable predictions. First, species that can learn more effectively should, on average, better conserve genetic diversity than species of less capable learners living in populations of similar size. The second and connected prediction is that species that are “better learners” should have a lower risk of extinction. This prediction is in good agreement with a recent study showing that bird species with greater behavioral plasticity indeed have a lower risk of extinction^12^, even though there are also likely other reasons responsible for this connection, such as the likely ability of behavioral plasticity to reduce mortality rates. The third prediction. In regard to our findings, there are two types of excess mortality, one which individuals of a given species may learn to avoid, and second that members of that species cannot learn to avoid or escape. Our findings suggest that the first type of excess mortality should reduce genetic diversity to a smaller degree than the second type, even if they cause the same increase in mortality rates. The analogous predictions can also be made with regard to immunological memory or any process reducing mortality by a systematic reactive mechanism.

Overall, our results show that the conventional view considering genetic drift as independent of underlying species behavior is incomplete and that genetic drift may be affected by common processes such as learning or immunological memory even if overall population-level mortality rates remain the same. Furthermore, we have shown that the level of protection against genetic drift varies in different situations, suggesting that loss of genetic diversity by genetic drift is a more complex issue than previously thought. We hope that these findings will make existing models of evolution more precise and could prove useful in a variety of topics, including the development of effective species conservation strategies, studies of the evolutionary past as well as the evolutionary future.

## Supplementary Information

Supplementary Information.

## Data Availability

The computer code used to generate results reported in this article can be accessed at https://github.com/PeterLenart/Learning_Mitigates_Genetic_Drift.
