# Supplementary material for: Learning mitigates genetic drift
Source: Sci Rep. 2022 Nov 27;12:20403. doi: 10.1038/s41598-022-24748-8 (PMC9701794; doi:10.1038/s41598-022-24748-8)
Supplement: Supplementary file 1 — Supplementary Information. [file 41598_2022_24748_MOESM1_ESM.pdf]

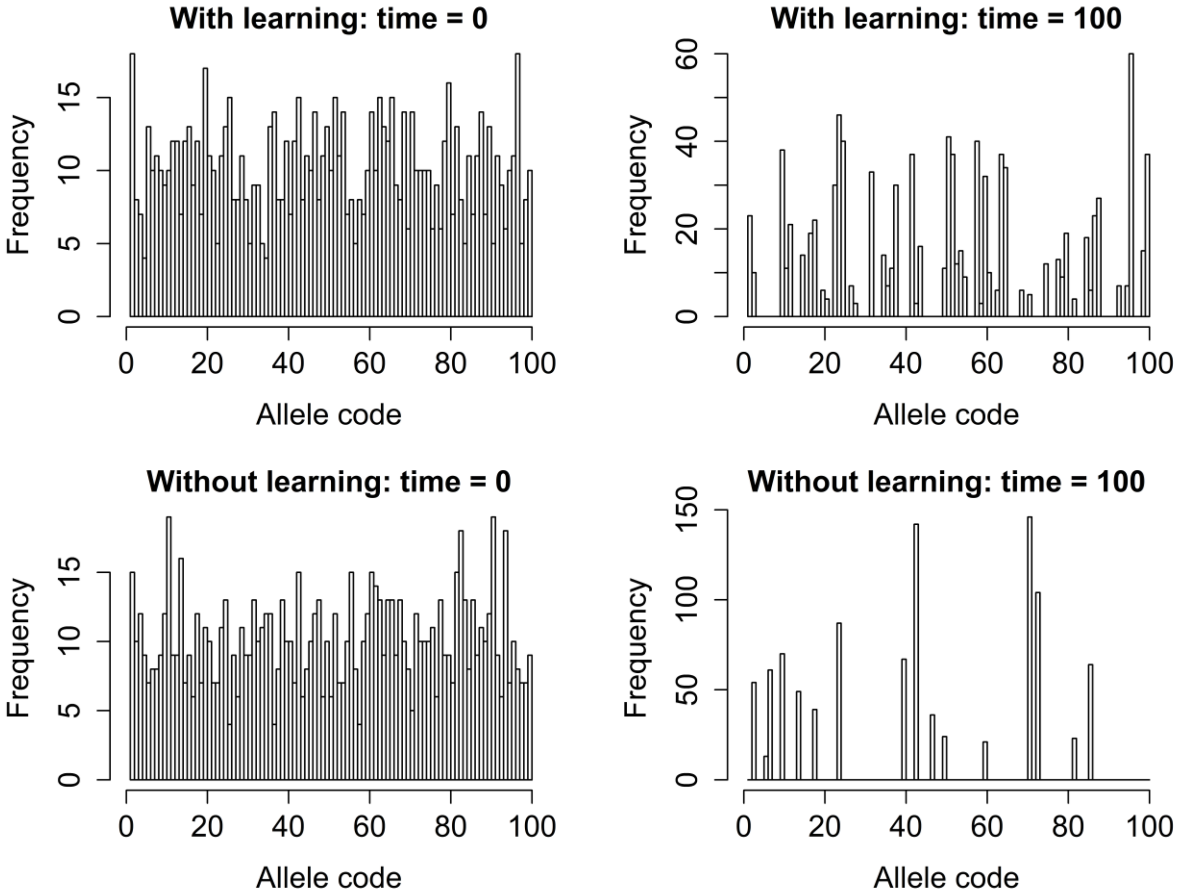

Supplementary figure S1: Learning slow downs loss of genetic diversity caused by genetic drift. The figure shows the changes in frequency of individual alleles after 100 time steps in a learning and non-learning scenario. In this single simulation run, prey populations consisted of 1000 individuals at the beginning of each step. Individuals killed by predators were replaced at the end of each step through *the* reproduction of surviving individuals (Methods). Predators consumed 500 prey individuals per timestep in both learning and non-learning scenarios to allow for a fair comparison. The upper bound on the probability to avoid or escape predator attack was set to 99%.

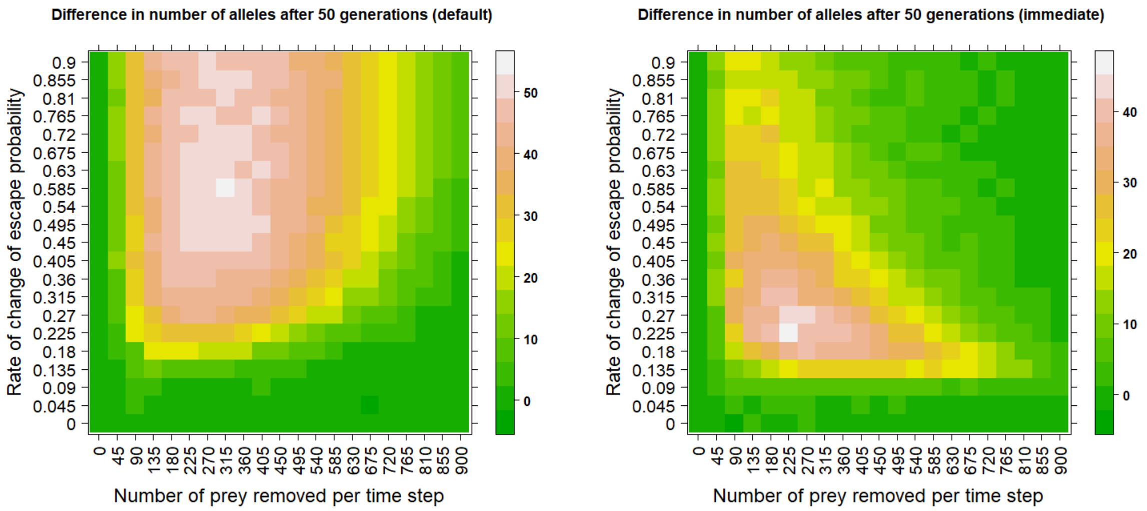

Supplementary figure S2: Decelerating learning produces results qualitatively identical to accelerating learning. The figure shows two heatmaps generated in simulations using the decelerating rate of learning. In the left panel, learning occurs in the default setting, i.e., at the end of every timestep. In the right panel, prey learns instantly after every unsuccessful predator attack. Predators kill the same number of individuals in both learning and non-learning scenarios to allow for a fair comparison. The upper bound on the probability of avoiding or escaping a predator attack is set to 0.99. The values in the heatmap are averages from 10 simulation runs.
